# Vitamin C Intake Reduces the Cytotoxicity Associated with Hyperglycemia in Prediabetes and Type 2 Diabetes

**DOI:** 10.1155/2013/896536

**Published:** 2013-07-25

**Authors:** Silvia Isabel Rech Franke, Luiza Louzada Müller, Maria Carolina Santos, Arcênio Fishborn, Liziane Hermes, Patrícia Molz, Camila Schreiner Pereira, Francisca Maria Assmann Wichmann, Jorge André Horta, Sharbel Weidner Maluf, Daniel Prá

**Affiliations:** ^1^PPG em Promoção da Saúde, Universidade de Santa Cruz do Sul, Avenida Independência, 2293, Sala 4206, 96815-900 Santa Cruz do Sul, RS, Brazil; ^2^Curso de Nutrição/DEDFIS, Universidade de Santa Cruz do Sul, Santa Cruz do Sul, RS, Brazil; ^3^Serviço de Genética Médica, Hospital de Clínicas de Porto Alegre, Porto Alegre, RS, Brazil

## Abstract

Hyperglycemia leads to the formation of free radicals and advanced glycation end-products (AGEs). Antioxidants can reduce the level of protein glycation and DNA damage. In this study, we compared the levels of vitamin C intake, which is among the most abundant antioxidants obtained from diet, with the levels of fasting plasma glucose (FPG), glycated hemoglobin (A1C), DNA damage, and cytotoxicity in prediabetic subjects and type 2 diabetic subjects. Our results indicated that there was no significant correlation between FPG or A1C and DNA damage parameters (micronuclei, nucleoplasmic bridges, and nuclear buds). FPG and A1C correlated with necrosis (*r* = 0.294; *P* = 0.013 and *r* = 0.401; *P* = 0.001, resp.). Vitamin C intake correlated negatively with necrosis and apoptosis (*r* = −0.246; *P* = 0.040, and *r* = −0.276; *P* = 0.021, resp.). The lack of a correlation between the FPG and A1C and DNA damage could be explained, at least in part, by the elimination of cells with DNA damage by either necrosis or apoptosis (cytotoxicity). Vitamin C appeared to improve cell survival by reducing cytotoxicity. Therefore, the present results indicate the need for clinical studies to evaluate the effect of low-dose vitamin C supplementation in type 2 diabetes.

## 1. Introduction

Diabetes mellitus (DM) includes a group of diseases that are characterized mainly by high levels of serum glucose (hyperglycemia) and a deficiency in or resistance to the action of the hormone insulin. There are approximately 200 million diabetics worldwide, and type 2 DM (DM2) accounts for 90–95% of all DM cases. DM2 is a very relevant pathology because of its high prevalence and related complications, including macrovascular (cardiovascular disease and ischemic encephalopathy), microvascular (coagulation dysfunction, nephropathy, and neuropathy), and biochemical (e.g., dyslipidemia) disturbances [1].

Clinical and experimental evidence suggests that DM complications are associated with metabolic disturbances that result mainly from hyperglycemia. Advanced glycation end-products (AGEs) are formed by a nonenzymatic reaction between glucose and basic amino acids, and their level is directly correlated with serum glucose levels [2]. The serum AGE level is a marker of late DM complications [3]. Glucose self-oxidation also leads to free radicals and oxidative stress formation, with the latter occurring when the concentration of free radicals is higher than the antioxidant capacity [4]. Oxidative stress is among the main causes of DM progression due to cell and tissue injury [1]. Glycated hemoglobin (A1C) is an altered form of hemoglobin that is produced by the action of AGEs, and it is considered to be a good marker of the average level of serum glucose over the previous weeks; A1C was shown to correlate with an increased risk of DM complications [2].

Antioxidant defenses comprise endogenous and exogenous enzymatic and nonenzymatic mechanisms. Vitamin C is a key exogenous nonenzymatic antioxidant that is found in high concentrations in serum and within cells. Vitamin C is an enzymatic cofactor and antioxidant that is capable of shifting between its oxidized and reduced forms by electron donors and that protects, at the intracellular level, DNA, proteins, and lipids against oxidative stress [5].

This study aimed to evaluate the relationship between the dietary intake of vitamin C, which is the main antioxidant in human blood, and the levels of A1C and primary DNA damage, which are two markers of oxidative stress, in prediabetic and DM2 subjects.

## 2. Materials and Methods

We evaluated the extent of DNA damage in whole blood samples collected from 70 prediabetic and DM2 subjects. All of the subjects were enrolled in the Ambulatory “Serviço Integrado de Saúde” of the University of Santa Cruz do Sul or were attending the Brazilian “Family Health Strategy,” both in Santa Cruz do Sul, RS, Brazil. The study protocol was approved (CAAE: 03981212.8.0000.5343) by the internal human experimentation ethics committee of the University of Santa Cruz do Sul, and all of the subjects gave written informed consent for their participation. The code of ethics of the World Medical Association (Declaration of Helsinki) was followed throughout the study.

Peripheral blood samples from all of the subjects were collected during the morning. Subjects were asked to fast and rest for an 8 h period before blood sampling, and only those who followed this recommendation were included in the study. Blood samples were immediately processed for fasting plasma glucose (FPG), A1C, DNA damage, and cytotoxicity levels. First, a blood subsample was centrifuged to obtain serum to measure the fasting glucose level. Second, another blood subsample was mixed with ethylenediaminetetraacetic acid for A1C determination. Third, a blood subsample was mixed with heparin for the cytokinesis-blocked micronucleus cytome assay (CBMN Cyt) analysis. The biochemical evaluations were conducted and the slides for the comet assay were prepared immediately after collection. FPG was measured using an enzymatic-spectrophotometric method, and A1C was measured using an HPLC method with the Bio-Rad Variant II Turbo Hemoglobin Testing System (Bio-Rad Laboratories, Hercules, CA, USA). Equipment, reagents, standards, and protocols for evaluating fasting plasma glucose (FPG) and A1C were, respectively, supplied by Biosystems S.A. (Barcelona, Spain) and Bio-Rad Laboratories, Hercules, CA, USA. 

For the CBMN Cyt analysis, samples were sent to the Cytogenetics Laboratory of the Porto Alegre Clinics Hospital. The CBMN Cyt measurement was performed according to the method described by Fenech and Morley [6] and as adapted by Maluf [7]. Blood samples (0.5 mL) were placed in 5 mL of culture medium containing RPMI 1640 supplemented with 20% fetal bovine serum and 2% phytohemagglutinin. The culture flasks were incubated at 37°C for 44 h, then cytochalasin B (final concentration 6 *μ*g/mL) was added, and the resulting suspensions were incubated for another 28 h. Thereafter, cell suspensions were treated with a hypotonic agent (KCl) and fixed in a 3 : 1 solution of acetic acid and methanol. Drops of the cell suspension were then placed on microscope slides (at least 2 per individual) and stained with Giemsa. The analysis was completed according to the standard criteria for CBMN Cyt [8]. Two thousand binucleated lymphocytes were analyzed per individual to assess the frequency of micronuclei (MN), nuclear buds (NBUD), nucleoplasmic bridges (NPB), apoptotic cells, and necrotic cells. The results are expressed as per 1000 cells.

The intake of vitamin C was determined using the Virtual Nutri 1.0 (São Paulo, SP, Brazil) software following the procedures described by Prá et al. [9]. In short, the habitual diet was evaluated on 3 nonconsecutive days (2 weekdays and 1 weekend day) using a validated questionnaire that contains open questions about typical food intake at each meal. Home measures were presented during the interview to aid in the interpretation of the amount of food ingested. 

Pearson's and Spearman's correlations and nonlinear curve-fitting tests were used to evaluate correlations between fasting glucose, A1C, DNA damage, and vitamin C. Statistical evaluations were performed and graphs were plotted using GraphPad Prism 4.0 (San Diego, CA, USA). The level of statistical significance was set at *P* < 0.05.

## 3. Results

Table 1 presents the characteristics of the study subjects. Most of the study subjects were female and most were more than 40 years old. Regarding the intake of vitamin C (Table 2), the prevalence of deficiency was 54.2% for men and 34.8% for women. The minimum and maximum mean intakes were 5 and 290 mg/day, respectively. Age correlated with FPG (*r* = 0.307; *P* = 0.030) and A1C (*r* = 0.428; *P* = 0.002). FPG correlated with A1C (*r* = 0.631; *P* < 0.001).

**Figure 1:**
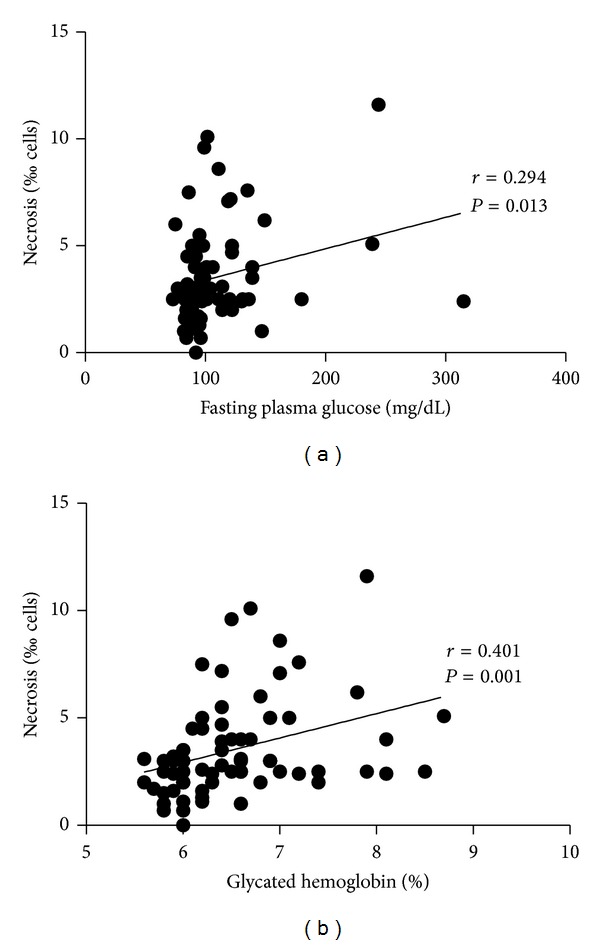
Correlations between fasting plasma glucose (a), glycated hemoglobin (b), and necrosis in prediabetic and type 2 diabetic adult subjects (*n* = 70). *r* and *P*: correlation coefficient and level of significance, respectively, according to the Spearman correlation test.

**Figure 2:**
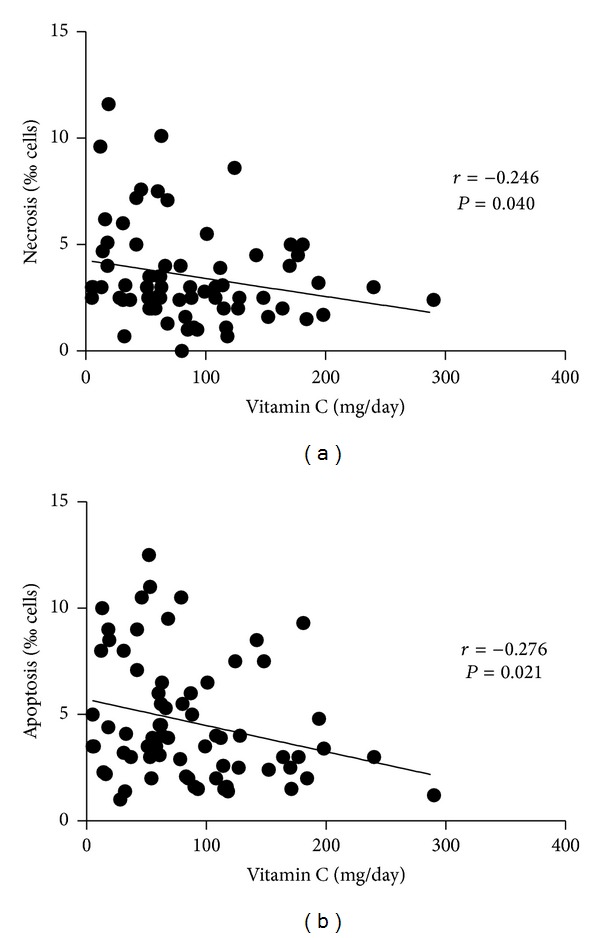
Correlations between vitamin C intake, necrosis (a), and apoptosis (b) in prediabetic and type 2 diabetic adult subjects (*n* = 70). *r* and *P*: correlation coefficient and level of significance, respectively, according to the Spearman correlation test.

**Figure 3:**
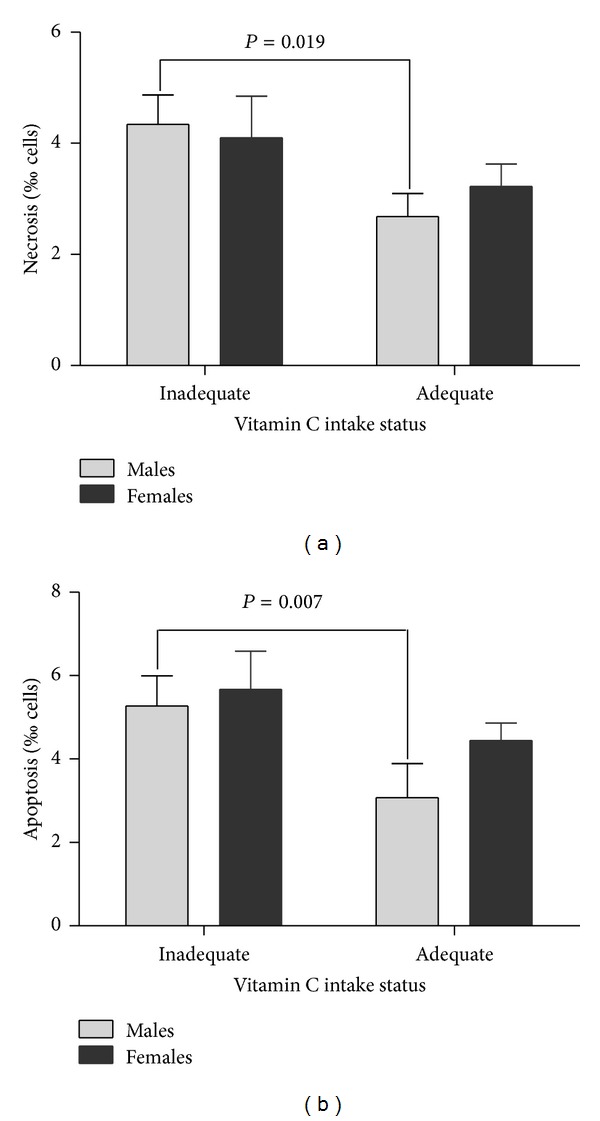
Necrosis (a) and apoptosis (b) according to adequate vitamin C intake status and sex in prediabetic and type 2 diabetic adult subjects (*n* = 70). Values are presented as the mean ± standard error of the mean. *P*: level of significance according to the Mann-Whitney *U* test. Adequate vitamin C intake was based on the estimated average requirement [10]. For clarification, see Table 2.

No significant correlations between FPG or A1C and DNA damage parameters (micronuclei, nucleoplasmic bridges, and nuclear buds) were observed. FPG and A1C correlated with necrosis (Figure 1; *r* = 0.294; *P* = 0.013 and *r* = 0.401; *P* = 0.001, resp.). Vitamin C intake correlated negatively with necrosis and apoptosis (Figure 2; *r* = −0.246; *P* = 0.040, and *r* = −0.276; *P* = 0.021, resp.). No significant correlation was observed between vitamin C intake and the DNA damage parameters (micronuclei, nucleoplasmic bridges, and nuclear buds). Necrosis and apoptosis were lower in subjects with an adequate intake of vitamin C in relation to those with an inadequate intake, but only for men (Figure 3). Vitamin C intake did not correlate significantly with A1C (*r* = −0.175; *P* = 0.148).

The nucleoplasmic bridges correlated with nuclear buds (*r* = 0.278; *P* = 0.020). Necrosis correlated with apoptosis (*r* = 0.624; *P* < 0.001).

## 4. Discussion

There is growing evidence that DM is linked to oxidative stress. Oxidative stress has been shown to be involved in many of the micro- and macrovascular complications that are associated with DM. DNA damage is among the well-known molecular effects of oxidative stress, and DNA damage increase has been observed in many chronic diseases with increased oxidative stress. The molecular evidence of increased DNA damage in white blood cells of diabetics is somewhat controversial, despite that changes in the DNA repair capacity of such cells are well documented [11–13].

Several studies have shown that vitamin C can improve metabolic dysfunctions associated with DM through, among other mechanisms, its antioxidant potential [14, 15]. It is well documented that vitamins are also capable of affecting DNA damage at different levels, including inhibiting damage formation, facilitating damage removal by DNA repair, and/or promoting cell death of the damaged cells through necrosis or apoptosis [16]. On the other hand, there are few studies in the literature (e.g., [17]) that link vitamins to DNA damage and cytotoxicity.

The mean vitamin C intake of the subjects in the present study (Table 2) was approximately the current estimated average requirement (EAR), which represents the adequate intake level and is used to evaluate nutrient intake for groups and individuals, according to dietary reference intake (DRI) [10]. A large portion of the cohort had inadequate intake of vitamin C, which was higher for men (>50% and <60% for men versus >30% and <40% for women). The vitamin C intakes of most of the individuals were lower than the 90–100 mg per day level recommended by Carr and Frei [18] for chronic disease risk reduction in nonsmoking men and women. The maximum intake observed among the studied subjects was approximately 300 mg/day, which is substantially lower than the 2000 mg/day level set by the Institute of Medicine (IOM) [10] as the maximum intake for healthy men and women. Therefore, the intake of all of the subjects was much lower than the level at which health risks start to occur.

FPG and A1C were correlated with age, as has been observed in previous studies [19, 20]. No correlations between DNA damage endpoints and age, FPG, or A1C were observed. Several studies have shown that there is no increase in DNA damage in DM2 [21, 22]. Other studies have indicated that an increase in micronuclei will not likely occur in early DM2 [23], but only in uncompensated DM2 (i.e., A1C > 8%) [24]. In our study, only 4 (approximately 6%) out of the 70 subjects had uncompensated DM2 (A1C > 8.0%); therefore, it was not likely that the sample would present an increase in chromosomal DNA damage. Until recently, it was not clear why individuals with early DM2 do not exhibit increased chromosomal DNA damage even though large levels of oxidative stress have been reported by several authors. It is likely that this effect might occur as a result of the increase in the frequency of necrosis and apoptosis (cytotoxicity) observed in early DM2. Human studies and those of rodent models of DM2 have indicated an increased frequency of necrosis and apoptosis [25]. We also observed an increase in apoptosis and necrosis in prediabetes [23]. There are two possible explanations for the cytotoxicity observed in DM2: (i) glucose self-oxidizes and generates oxidative stress that damages different cell compartments, possibly leading to cell death [26], and (ii) iron participates in several redox reactions and can generate a large amount of reactive oxygen species that could also mediate cell death. There are several studies that show a link between iron and DM2 risk, for example, due to the high cycling of hemoglobin arising from the short half-life of red blood cells [23]. However, the exact link between cytotoxicity and DNA damage in DM2 remains to be elucidated.

Vitamin C reduces the levels of oxidative stress. Additionally, vitamin C might have roles in DNA repair, reducing the extent of DNA damage [27]. There is evidence that diabetics have depleted serum levels of vitamin C and, therefore, require intake levels that are slightly higher than those recommended for non-diabetic individuals. Choi et al. [15] evaluated the relationship between fasting plasma vitamin C, lymphocyte primary DNA damage (comet assay), and A1C in 427 DM2 individuals and observed a similar negative correlation between DNA damage and serum vitamin C. Because dietary intake was not evaluated by Choi et al. [15], the potential use of their data for dietary recommendations is limited. In contrast, in our study, we showed that cytotoxicity decreases when the EAR is met, but only among men. The EAR for men is higher (75 mg vitamin C per day) than for women (60 mg vitamin C per day). Therefore, the present results reflect a low intake of vitamin C in our study population, and the results indicate that intake levels that are slightly higher than the DRI for vitamin C are linked to a reduced risk of DM2-associated complications, at least in normal dietary conditions. The present results also indicate the need for clinical studies that evaluate the effect of low-dose vitamin C supplementation in DM2. It is important to highlight that many previous studies have used large vitamin C dosages; under these conditions, vitamin C was shown to act as a prooxidant.

The present study has several limitations as follows: (i) we did not include control subjects (individuals without prediabetes or DM2), and we had only a few individuals with uncompensated DM2, which is different than similar studies and could represent a limitation in comparing the results; (ii) the sample size was small, which did not allow for the generation of dietary recommendations; (iii) we did not measure the plasma level of vitamin C but instead measured the dietary level, which could be subject to recall errors; (iv) we did not consider the different doses and types of hypoglycemiant drugs that were taken by the patients who were under medical treatment; and (v) we did not evaluate the mechanism by which DM2 induces cytotoxicity and how vitamin C modulates that cytotoxicity. Despite these limitations, the correlation observed between vitamin C intake and cytotoxicity is important when designing interventions to reduce the complications that are associated with hyperglycemia in DM2.

## Figures and Tables

**Table 1:** Characteristics of the studied cohort of prediabetic and type 2 diabetic adult individuals (*n* = 70).

Sex (male/female)	24/46					
	Average ± SD	Minimum	Maximum	P25	P50	P75
Age (years)	51.9 ± 10.9	28	76	46	53	60
Fasting serum glucose (mg/dL)	109.3 ± 39.5	73	315	89	97	119
Glycated hemoglobin (%)	6.5 ± 0.70	5.7	8.7	6.0	6.4	6.9
Micronuclei (‰ cells)	0.58 ± 0.95	0.0	7.0	0.0	0.5	1.0
Nucleoplasmic bridges (‰ cells)	1.55 ± 1.47	0.0	11.0	0.5	1.0	2.0
Nuclear buds (‰ cells)	1.02 ± 1.03	0.0	7.0	0.5	1.0	1.0
Necrosis (‰ cells)	3.54 ± 2.30	0.0	12.0	2.0	3.0	4.5
Apoptosis (‰ cells)	4.66 ± 2.85	1.0	13.0	2.5	4.0	6.5

P: percentile.

**Table 2:** Assessment of the intake of vitamin C according to sex in the studied cohort of prediabetic and type 2 diabetic adult individuals (*n* = 70).

	Vitamin C intake (mg/day)											
	EAR	Mean ± SD	Percentile of usual intake distribution									Assessment comments
			10th	20th	30th	40th	50th	60th	70th	80th	90th	
Men (*n* = 24)	75	80.5 ± 65.7	9.6	18.5	43.9	53.5	61.9	89.7	103.5	116.9	174.3	Prevalence of inadequacy is >50% but <60%
Women (*n* = 46)	60	88.5 ± 57.3	18.6	34.0	53.7	63.1	73.2	87.2	113.6	145.4	181.5	Prevalence of inadequacy is >30% but <40%

SD: standard deviation; EAR: estimated average requirement [10].
